# Nitrogen Source Dependent Changes in Central Sugar Metabolism Maintain Cell Wall Assembly in Mitochondrial Complex I-Defective *frostbite1* and Secondarily Affect Programmed Cell Death

**DOI:** 10.3390/ijms19082206

**Published:** 2018-07-28

**Authors:** Anna Podgórska, Monika Ostaszewska-Bugajska, Agata Tarnowska, Maria Burian, Klaudia Borysiuk, Per Gardeström, Bożena Szal

**Affiliations:** 1 Institute of Experimental Plant Biology and Biotechnology, Faculty of Biology, University of Warsaw, I. Miecznikowa 1, 02-096 Warsaw, Poland; m.ostaszewska@biol.uw.edu.pl (M.O.-B.); atarnowska@biol.uw.edu.pl (A.T.); mburian@biol.uw.edu.pl (M.B.); k.borysiuk@biol.uw.edu.pl (K.B.); szal@biol.uw.edu.pl (B.S.); 2 Umeå Plant Science Centre, Department of Plant Physiology, Umeå University, SE-90187 Umeå, Sweden, per.gardestrom@umu.se

**Keywords:** cell wall synthesis, complex I defect, *frostbite1*, mitochondrial mutant, NDUFS4, necrosis, sugar catabolism, sugar signaling, programmed cell death, reactive oxygen species

## Abstract

For optimal plant growth, carbon and nitrogen availability needs to be tightly coordinated. Mitochondrial perturbations related to a defect in complex I in the *Arabidopsis thaliana* *frostbite1* (*fro1*) mutant, carrying a point mutation in the 8-kD Fe-S subunit of NDUFS4 protein, alter aspects of fundamental carbon metabolism, which is manifested as stunted growth. During nitrate nutrition, *fro1* plants showed a dominant sugar flux toward nitrogen assimilation and energy production, whereas cellulose integration in the cell wall was restricted. However, when cultured on NH_4_^+^ as the sole nitrogen source, which typically induces developmental disorders in plants (i.e., the ammonium toxicity syndrome), *fro1* showed improved growth as compared to NO_3_^−^ nourishing. Higher energy availability in *fro1* plants was correlated with restored cell wall assembly during NH_4_^+^ growth. To determine the relationship between mitochondrial complex I disassembly and cell wall-related processes, aspects of cell wall integrity and sugar and reactive oxygen species signaling were analyzed in *fro1* plants. The responses of *fro1* plants to NH_4_^+^ treatment were consistent with the inhibition of a form of programmed cell death. Resistance of *fro1* plants to NH_4_^+^ toxicity coincided with an absence of necrotic lesion in plant leaves.

## 1. Introduction

Plants are autotrophic organisms that use assimilated nitrogen and carbon for the biosynthesis of proteins and other organic compounds in order to fulfil the developmental needs of their organs. It must be noted that the assimilation of nitrogen is one of the most energy-consuming cellular processes for plants. Indeed, the reduction of nitrate (NO_3_^−^) to ammonium (NH_4_^+^) and its incorporation into amino acids consumes the equivalent of 12 ATP molecules [1,2]. Like all living organisms, plants require energy (in the form of ATP) and reductants (mainly NADH and NADPH) for their maintenance. Plant mitochondria carry out the final step of respiration to integrate sugar catabolism with ATP production. Therefore, mitochondria drive metabolism throughout the cell, since they can regulate energy and redox balance [3,4,5]. Furthermore, the central metabolic position of mitochondria, and their key roles in bioenergetics, mean that they are ideally placed to act as sensors and integrators of the biochemical status of the cell. On the other hand, a close and active communication between mitochondria and other organelles and the nucleus (retrograde signaling) exists to adjust correct metabolic function of plants in response to different environmental conditions [6,7]. Additionally, a significant role in the response of mitochondria to stress conditions has been proposed for reactive oxygen species (ROS), which can be very effective signaling molecules [8,9]. Thus, mitochondrial retrograde signals can mediate diverse developmental processes, from growth regulation to programmed cell death (PCD).

The classical mitochondrial electron-transport chain (mtETC) is composed of four respiratory oxidoreductases (complexes I–IV), which couple redox energy recycling with ATP synthesis catalyzed by the ATP synthase complex (complex V) [10]. The main entry point for electrons to the mtETC is complex I, which functions as an NADH dehydrogenase. Complex I oxidizes matrix NADH, which can be supplied primarily by the tricarboxylic acid (TCA) cycle, by glycine decarboxylation or cytosolic NADH generated mainly during glycolysis and further shuttled into mitochondria by the oxaloacetate (OAA)-malate valve. Loss of complex I activity in mutant plants lowers the efficiency of oxidative phosphorylation by more than 30% [11]. Nevertheless, the activity of specific plant type II dehydrogenases allows electrons from NAD(P)H to enter the mtETC, which enables mutants to survive without a functional complex I [12,13]. Complex I, which is composed of many subunits, is the largest transmembrane, proton-pumping complex in the mtETC. Mutations in any of these subunits can severely hinder or even inhibit complex I assembly or activity. To date, only four mutants in mitochondrially encoded subunits have been characterised—cytoplasmic male sterility in tobacco (*Nicotiana sylvestris*), CMSII, [14,15,16], non-chromosomal stripe in maize (*Zea mays*), NCS2, [17,18], mosaic phenotype (MSC16) in cucumber (*Cucumis sativus*) [19,20]. In addition, nuclear-gene encoded mutants having defects in complex I have been identified in *Arabidopsis*, e.g., *ndufs4* and *ndufv1* [11,21]. Moreover, there are several *Arabidopsis thaliana* complex I mutants that are defective in other complex I-connected subunits, including *ca1ca2* [22] and *atcib22* [23], or are connected to splicing factors such as *otp43* [24], *css1* [25], *nMat2* [26], *rug3* [27], *mterf15* [28], and *bir6* [29], as well as an *N. sylvestris* mutant, NMS1 [30,31]. In the last decade, several of these complex I mutants have been characterized (reviewed by [32,33]).

All mutants with dysfunction or loss of complex I exhibit reorganized respiratory metabolism, which may affect their redox and energy status. MSC16 plants showed lower NAD(P)H availability [34] and lower respiratory rates, which resulted in lower ATP contents [20,35]. Similarly, the NMS1 and NCS2 mutants showed reduced respiratory capacity but no data about their adenylate or nucleotide status is available [17,31]. Even though *ndufs4* showed normal respiratory capacity, the mutant produced only limited amounts of ATP [11]. The exception is the CMSII mutant, which had a higher content of adenylates and NAD(P)H [34,36], concomitantly with unchanged respiratory fluxes [16,29]. Overall, research using complex I mutants indicates that complex I defects in plants are compensated by reorganization of respiration, although oxidative phosphorylation rates are not fully restored, and most mutant plants are energy deficient. Because of their altered metabolic status, most complex I mutants examined so far showed retarded growth and developmental disorders, in comparison to wild-type (WT) plants. Moreover, a defect in the mtETC often correlates with the occurrence of oxidative stress [11,36,37], and mitochondria were mainly highlighted in these mutants as a primary source of the observed higher rates of ROS generation [37]. Furthermore, a reduced complex I abundance was also found to affect mitochondrial biogenesis. Mutants plants were characterized by altered mitochondrial transcription, translation, and showed altered protein uptake capacities [27,28,38,39].

Interestingly, many complex I mutants apparently have high tolerance to stress conditions. In CMSII plants, higher tolerance to ozone and to the tobacco mosaic virus was detected [16,40,41,42]. The MSC16 mutant showed an increased resistance to chilling stress and high irradiance conditions [35,38]. In NCS2 plants, improved tolerance to oxidative stress was observed, which limited initiation of PCD [43,44]. In a study of several types of stress (drought, osmotic, chilling, freezing, paraquat, NaCl, H_2_O_2_), *ndufs4* mutant plants showed improved resistance to abiotic stress conditions in comparison to the WT [11,45]. Similarly, the *nMat22* mutant showed improved tolerance to ethanol treatment [23] and *bir6* was resistant to salt and osmotic stress [29]. Another complex I mutant was discovered by chance when looking for genes involved in stress signal transduction in an ethyl methanesulfonate-mutagenized population under different stress conditions and was named *frostbite1* (*fro1*), because of its susceptibility to chilling temperatures [46]. It was shown that *fro1* plants had a single point mutation in the nuclear-encoded 18-kDa Fe–S subunit of complex I, which concerned a G-to-A change at an intron–exon junction at the start codon resulting in missplicing and a premature stop codon [46]. Consequently, the lack of NDUFS4 led to the disassembly of complex I [47]. Moreover, the *fro1* mutation reduced the expression of stress-inducible genes during chilling conditions, which impaired cold acclimation, whereby mutants also became sensitive to other stress factors like NaCl and osmotic stress [46]. In contrast to these responses, in our recent study, *fro1* plants showed improved resistance to ammonium nutrition [47].

Cultivation using NH_4_^+^ as the sole nitrogen source for many plants, including *Arabidopsis,* leads to severe toxicity symptoms known as the “ammonium syndrome” [48,49]. Ammonium regulates many physiological processes, ranging from mitosis and cell elongation to senescence and death; hence, ammonium availability may act as a major determinant of plant morphogenesis [50,51]. During NH_4_^+^ nutrition, nitrate reduction reactions catalyzed by nitrate reductase (NR) and nitrite reductase (NiR) are bypassed, resulting in a surplus of reductants in the cytosol and chloroplasts. Therefore, in terms of energy economy, NH_4_^+^ would seem to be a better source of nitrogen for plants, as its assimilation requires less energy than that for NO_3_^−^ [1,2]. However, plants cultured on NH_4_^+^ as a sole nitrogen source often exhibit serious growth disorders; still, despite many years of research concerning this phenomenon, the cause is still not well understood [52,53]. Plant mitochondria are a source of metabolites needed during NH_4_^+^ assimilation, particularly the TCA cycle, which is the origin of the necessary 2-oxoglutarate (2-OG) for amino acid synthesis [2,52,54]. Elevated activity of the TCA cycle during NH_4_^+^ nutrition increases mitochondrial matrix NADH production, which must be oxidized by the mtETC [55,56]. Therefore, ammonium nutrition may primarily affect plant mitochondria, since the increased load of redox equivalents to the mtETC and the consequent high respiratory capacity leads to elevated ROS levels in mitochondria [57]. 

Furthermore, use of the *fro1* mutant revealed that the combined effect of an impairment of complex I and NH_4_^+^ treatment not only affects mitochondrial functioning in plants, but also changes their extracellular metabolism [47]. It is known that higher cell wall stiffness in response to NH_4_^+^ nutrition, resulting from altered cell wall modifying enzyme activities, can restrict expansion growth of plant cells [58]. Thus, the aim of this study was to investigate the interplay between mitochondrial functioning and cell wall-related processes in response to NH_4_^+^ nutrition. To understand changes in the growth rate of *fro1* when cultured on NH_4_^+^, properties of cell walls and sugar metabolism were examined. Moreover, the role of plant mitochondria in retrograde signalling and PCD was analyzed.

## 2. Results

### 2.1. Characterization of fro1 Plants Cultured on Different Nitrogen Sources

**Figure 1:**
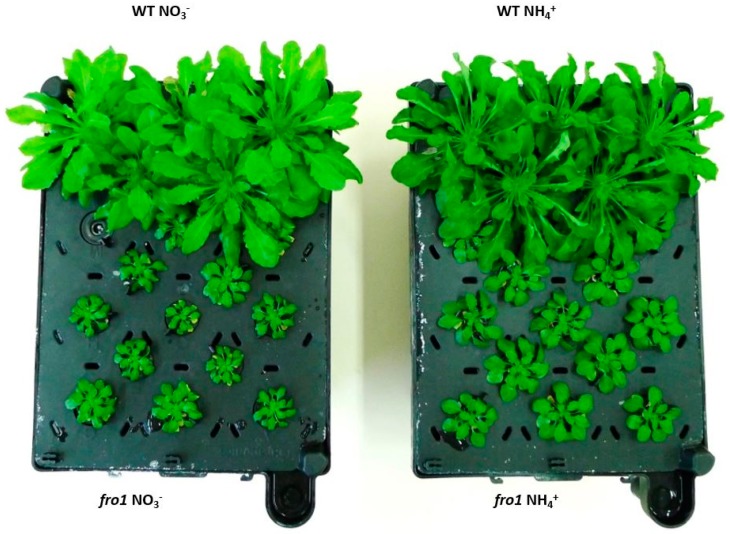
Phenotype of *frostbite1* (*fro1*) or wild-type (WT) *Arabidopsis* ecotype C24 plants cultured hydroponically for 8 weeks on nutrient medium containing either 5 mM nitrate (NO_3_^−^) or 5 mM ammonium (NH_4_^+^) as the only nitrogen source.

The consequences of limited ability to oxidize cellular oxidants in mutants carrying a point mutation in NDUFS4 (AT5G67590), affecting complex I assembly—*frostbite1* [46,47], on plant growth was observed under NH_4_^+^ and NO_3_^−^ (control) nutrition. During NO_3_^−^ assimilation, *fro1* plants showed strong growth retardation, compared to WT plants (*Arabidopsis thaliana,* ecotype C24) (Figure 1). However, under NH_4_^+^ nutrition *fro1* plants grew overall bigger rosettes than the same plants cultured in NO_3_^−^ conditions, while WT plants displayed growth inhibition in response to the NH_4_^+^ treatment (Figure 1) (similarly to a previous report by [47]).

### 2.2. Sugar Metabolism in fro1

**Figure 2:**
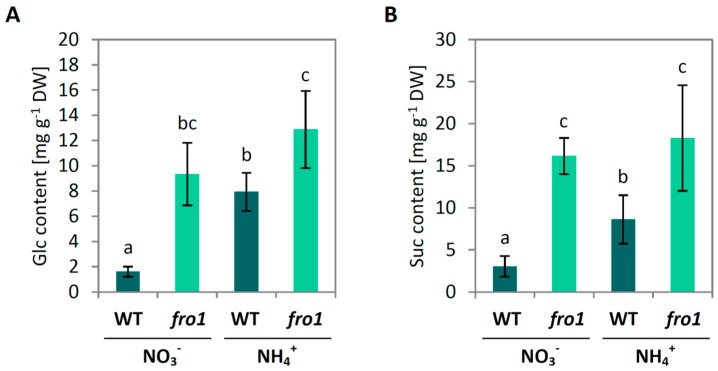

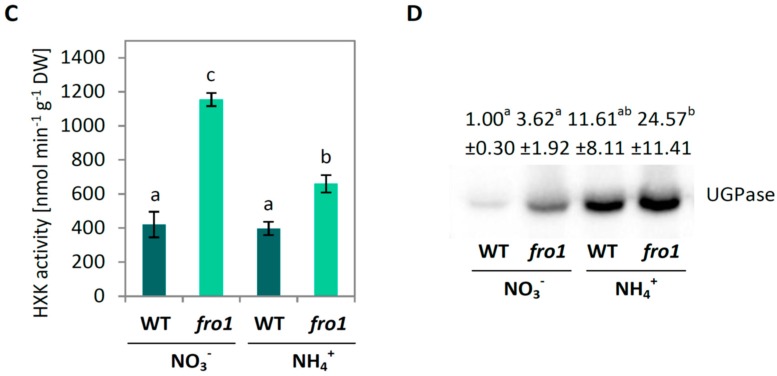
Polysaccharide metabolism in leaves of *frostbite1* (*fro1*) or wild-type (WT) *Arabidopsis* ecotype C24 plants cultured on NH_4_^+^ and NO_3_^−^ as the only nitrogen source. (**A**) Content of glucose (Glc) and (**B**) Sucrose (Suc); (**C**) activity of hexokinase (HXK); (**D**) protein level of UDP-glucose pyrophosphorylase (UGPase) in leaf tissue extracts. Representative blot is shown. Values are the mean ± standard deviation (SD) of three biological and two technical replicates. Means with different letters are significantly different (*p* < 0.05) by ANOVA followed by Tukey’s test.

We investigated whether the changes brought about by disabled function of mtETC in *fro1* are connected to changes in sugar metabolism. *Fro1* plants showed higher sucrose (Suc) and glucose (Glc) contents in leaf tissue when cultured on NO_3_^−^-containing medium as compared to WT plants. In contrast, growth on ammonium led to an increase in soluble sugar content in WT but not in *fro1* plants (Figure 2A,B). Hexokinase (HXK) activity was almost 3 times higher in *fro1* than in WT plants grown under NO_3_^−^ conditions. On the other hand, it remained unchanged in WT plants under NH_4_^+^ treatment, while it decreased in *fro1*, although it was still higher than in WT plants (Figure 2C). Protein level of UDP-Glc pyrophosphorylase (UGPase) was not statistically different between *fro1* and WT under control growth conditions but increased significantly more in *fro1* as compared to WT plants under NH_4_^+^ treatment (Figure 2D and Figure S1).

### 2.3. Analysis of Cell Size and Cell Wall Design in fro1

**Figure 3:**
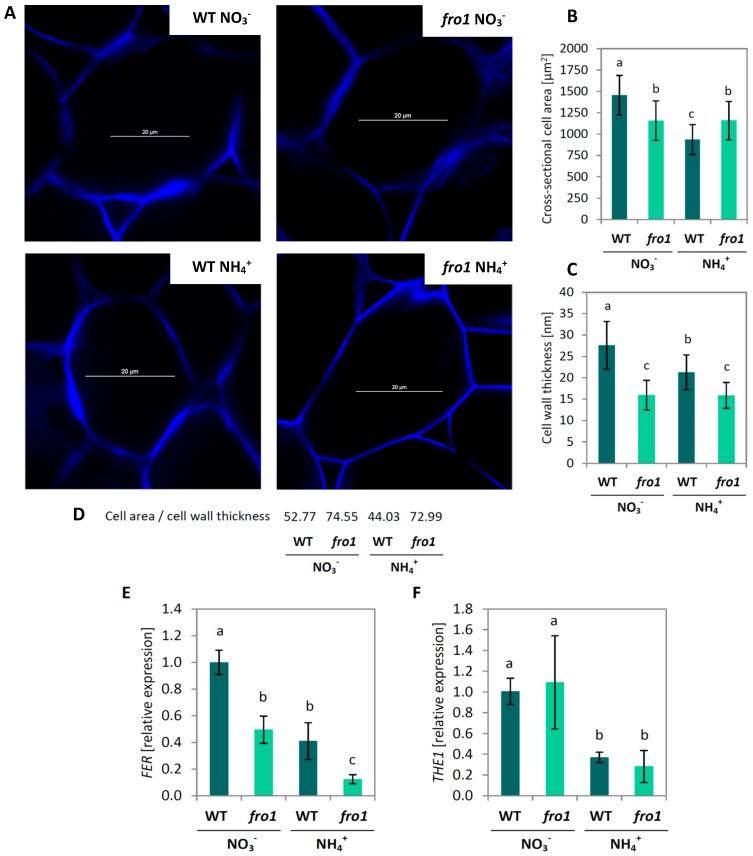
Cell wall characterization of leaf cells of *frostbite1* (*fro1*) or wild-type (WT) *Arabidopsis* ecotype C24 plants cultured on NH_4_^+^ and NO_3_^−^ as the only nitrogen source. (**A**) Cell wall visualization of individual palisade cells by Calcofluor White staining in CLSM (representative images are shown; scale bar = 20 µm); (**B**) cross-sectional cell area measured from 8 independent biological replicates; (**C**) cell wall thickness calculated from 10 independent images; (**D**) ratio of cell area to cell wall thickness; (**E**) transcript levels for *Feronia* (*FER*) and (**F**) *Thesseus1* (*THE1*) determined from three biological and two technical replicates. Values are the mean ± standard deviation (SD). Means with different letters are significantly different (*p* < 0.05) by ANOVA followed by Tukey’s test.

Cell wall in palisade cells was visualized using Calcofluor White staining (Figure 3A). As observed for changes in rosette size, *fro1* showed a smaller cell size under NO_3_^−^ nutrition than WT plants, as determined by the cross-sectional area of individual cells. Surprisingly, the cell area of *fro1* plants remained unchanged under NH_4_^+^ nutrition, while in WT plants it decreased by 35% (Figure 3B). To better understand the growth rate of *fro1* plants when cultured on NH_4_^+^, we analyzed cell wall properties. Cell wall thickness exhibited a similar trend as cell size (Figure 3C). However, the ratio of cell area to cell wall thickness was 40% higher in NO_3_^−^-treated *fro1* plants than in WT (Figure 3D). Ammonium nutrition led to its decrease only in WT plants, while in *fro1*, it was maintained at a similar level as under NO_3_^−^ nutrition (Figure 3D). Further, the correlation of cell wall assembly and cell wall sensing receptor-like kinases was determined [59]. Expression of *Feronia* (*FER,* AT3G51550), which is thought to be associated with cell wall-dependent regulation of cell elongation, was lower in *fro1* than in WT plants, while NH_4_^+^ treatment lowered the expression in both genotypes significantly (Figure 3E). The expression of *Thesseus1* (*THE1*, AT5G54380), a cell wall integrity sensor-kinase, was similar in *fro1*, as compared to WT plants under NO_3_^−^ nutrition, but it decreased about 70% during the NH_4_^+^ growth-regime in both genotypes (Figure 3F).

### 2.4. Cell Wall Composition in fro1

**Figure 4:**
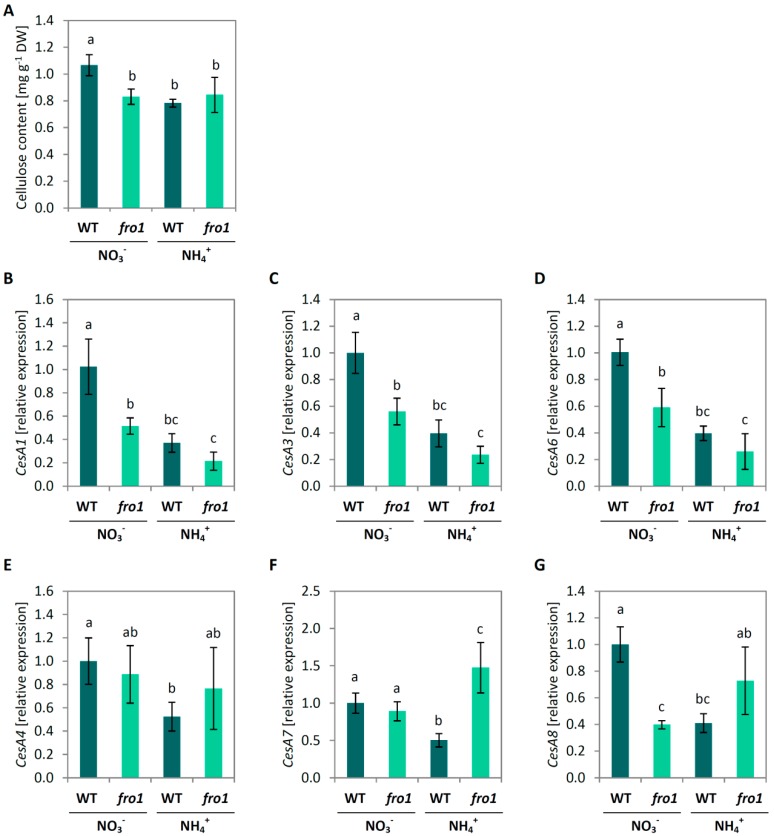
Cellulose metasbolism in leaves of *frostbite1* (*fro1*) or wild-type (WT) *Arabidopsis* ecotype C24 plants cultured on NH_4_^+^ and NO_3_^−^ as the only nitrogen source. (**A**) Cellulose levels measured in dried cell walls. Transcript level for (**B**) cellulose synthase (Ces) *A1*, (**C**) *CesA3*, (**D**) *CesA6*, (**E**) *CesA4*, (**F**) *CesA7*, (**G**) *CesA8*. Values are the mean ± standard deviation (SD) of three biological and two technical replicates. Means with different letters are significantly different (*p* < 0.05) by ANOVA followed by Tukey’s test.

The analysis of cell wall building components showed decreased cellulose incorporation in cell walls of *fro1*, compared to WT plants during NO_3_^−^ feeding (Figure 4A). However, NH_4_^+^ nutrition did not affect the cellulose content in *fro1* in contrast to WT plants, which exhibited a decrease in its level (Figure 4A). To explain those differences between genotypes, the expression of selected cellulose synthase (*CesA*) genes was examined. The genes *CesA1* (AT4G32410), *CesA3* (AT5G05170), and *CesA6* (AT5G64740) are specifically involved in cellulose synthesis during primary wall formation, while *CesA4* (AT5G44030), *CesA7* (AT5G17420)*,* and *CesA8* (AT4G18780) are involved in cellulose synthesis during secondary cell wall assembly [60]. The expression of all analyzed genes associated with cellulose synthesis in primary cell walls exhibited a similar pattern. *CesA1*, *CesA3*, and *CesA6* relative transcript level was about 50% lower in *fro1* than in WT plants under NO_3_^−^ nutrition; further, the treatment of plants with NH_4_^+^ led to decreases (by approx. 50%) in expression of these genes in both genotypes (Figure 4B–D). Transcript level of *CesA4* and *CesA7* was similar in *fro1* plants, while *CesA8* was about 60% lower than in WT plants during NO_3_^−^ nutrition (Figure 4E–G). All genes were down-regulated in WT plants in response to NH_4_^+^ nutrition, in contrast to NH_4_^+^-treated *fro1,* in which their expression was up-regulated, except for the gene *CesA4* whose expression remained similar to that under NO_3_^−^ growth conditions (Figure 4B–D).

**Figure 5:**
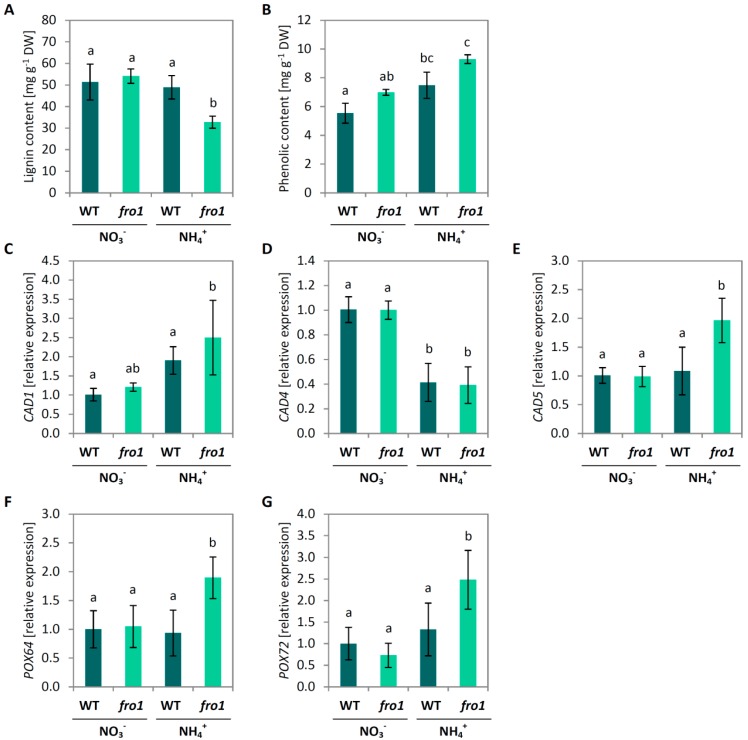
Lignin metabolism in leaves of *frostbite1* (*fro1*) or wild-type (WT) *Arabidopsis* ecotype C24 plants cultured on NH_4_^+^ and NO_3_^−^ as the only nitrogen source. (**A**) Lignin and (**B**) phenolic content in cell walls. Transcript level for (**C**) cinnamyl alcohol dehydrogenase (CAD) *1*, (**D**) *CAD4*, (**E**) *CAD5*, and peroxidases (POX) related to cell wall lignification: (**F**) *POX64* and (**G**) *POX72*. Values are the mean ± standard deviation (SD) of three biological and two technical replicates. Means with different letters are significantly different (*p* < 0.05) by ANOVA followed by Tukey’s test.

**Figure 6:**
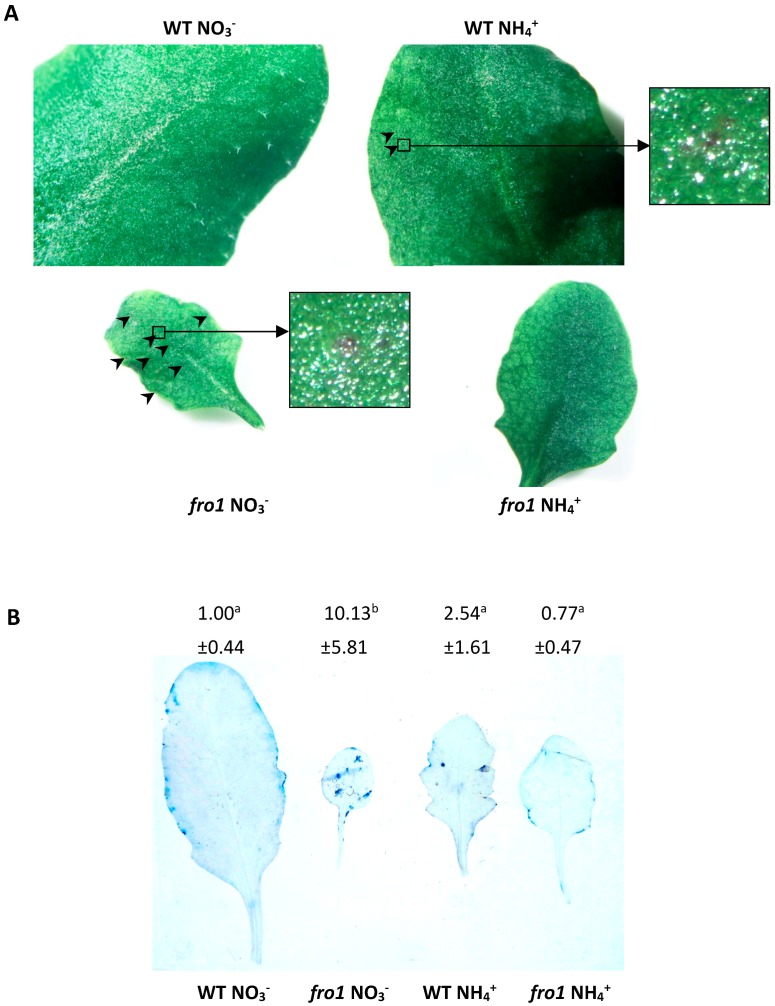
Visualization of lesions on leaves of *frostbite1* (*fro1*) or wild-type (WT) *Arabidopsis* ecotype C24 plants cultured on NH_4_^+^ and NO_3_^−^ as the only nitrogen source. (**A**) Appearance of necrosis on leaf blades indicated by arrows and (**B**) necrosis stained with trypan blue. Representative leaf blades from three independent plant cultures are shown. Means with different letters are significantly different (*p* < 0.05) by ANOVA followed by Tukey’s test.

Lignin, a highly cross-linked polymer, is formed to support the structure of the secondary cell wall in plants. The content of lignin in cell walls was similar in *fro1* and WT plants. Ammonium nutrition lowered lignification of cell walls in *fro1* plants but it did not influence lignin level in WT plants (Figure 5A). As lignin is composed of phenolic polymers, we analyzed total content of phenolics in the cell wall, and found it was higher in *fro1*, compared to WT plants under NO_3_^−^ nutrition. Ammonium nutrition caused further increase in phenolic levels in plants (Figure 5B). Primary genes involved in lignin biosynthesis are cinnamyl alcohol dehydrogenase genes (CAD) *CAD1* (AT1G72680), *CAD4* (AT3G19450), and *CAD5* (AT4G34230) [61]. The *fro1* mutant did not show significant changes in the expression of any of the analyzed *CAD* genes as compared to WT under the NO_3_^−^ growth regime (Figure 6C–E). In response to NH_4_^+^ nutrition, *CAD4* expression was inhibited by more than 50% in both genotypes, while *CAD1* and *CAD5* was up-regulated only in *fro1* plants (Figure 5C–E). Further, we analyzed the expression patterns of cell wall peroxidases (POX) related to cell wall lignification. The transcript level of *POX64* (AT5G42180) and *POX72* (AT5G66390) was unchanged between *fro1* and WT plants during NO_3_^−^ nutrition, but it showed an increase in *fro1* in response to NH_4_^+^ treatment (Figure 5F,G).

### 2.5. Analysis of Programmed Cell Death Markers in fro1

Visual examination of leaf blades of *fro1* or WT plants revealed the N source-dependent presence of lesions. In WT plants, ammonium caused emergence of few lesions. Conversely, in the case of *fro1* plants, lesions appeared in NO_3_^−^-supplied mutants and were absent in NH_4_^+^-treated plants (Figure 6A and Figure S3). Furthermore, trypan blue staining of leaves was performed to specifically indicate necrotic areas. Characteristic blue spot appearance on leaves revealed strong development of necrotic areas on leaves of *fro1* when grown on NO_3_^−^ (Figure 6B and Figure S4), while NH_4_^+^ nutrition induced the occurrence of some necrotic areas in WT plants, which could mostly not be identified in NH_4_^+^-grown *fro1*.

**Figure 7:**
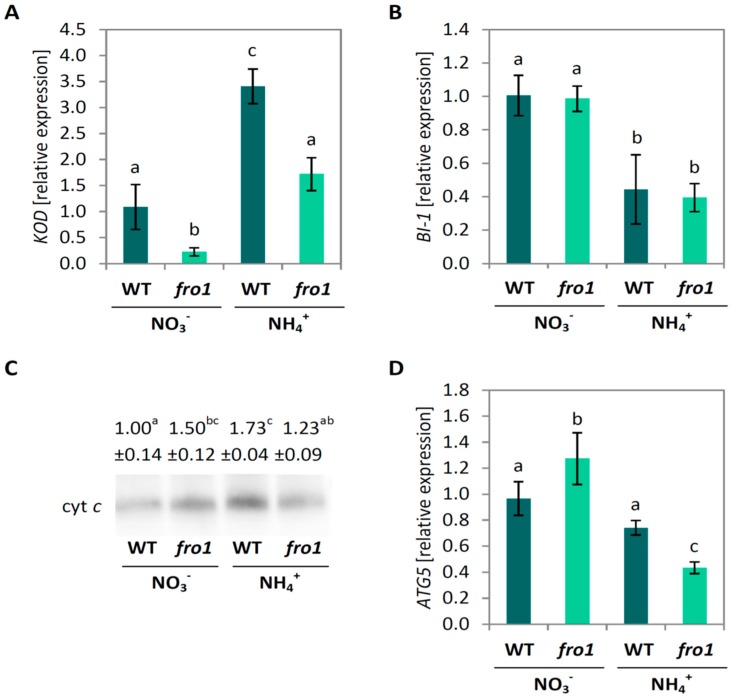
Programmed cell death markers in *frostbite1* (*fro1*) or wild-type (WT) *Arabidopsis* ecotype C24 plants cultured on NH_4_^+^ and NO_3_^−^ as the only nitrogen source. Transcript levels for (**A**) Kiss-of-death (*KOD*); (**B**) Bax inhibitor 1 (*BI-1*) genes; (**C**) Cytochrome *c* (cyt *c*) protein level in isolated mitochondria and (**D**) transcript level for autophagy 5 (*ATG5*) gene. Values are the mean ± standard deviation (SD) of three biological and two technical replicates. Means with different letters are significantly different (*p* < 0.05) by ANOVA followed by Tukey’s test. Representative blot is shown.

The marker gene for PCD called *Kiss-of-death* (*KOD*) [62] showed a lower expression in *fro1* plants under NO_3_^−^ nutrition and was up-regulated by NH_4_^+^ treatment in both genotypes (Figure 7A). At the same time, down-regulation of expression of *Bax inhibitor 1* (*BI-1*) (AT5G47120) under NH_4_^+^ nutrition was observed in both genotypes. The expression of *BI-1* was not changed in response to mtETC dysfunction (Figure 7B). Expression of the autophagy-related gene *ATG5* was induced in *fro1* during NO_3_^−^ nutrition as compared to WT. While the expression was unchanged in response to the NH_4_^+^ treatment in WT plants, in *fro1* the transcript level was approximately 60% lower (Figure 7D). Additionally, *fro1* plants showed 50% higher cytochrome *c* (cyt *c*) levels than WT under NO_3_^−^ conditions. Similar to previous observations in WT *Arabidopsis thaliana* [63], NH_4_^+^ nutrition led to approximately 70% higher cyt *c* level. The NH_4_^+^ treatment had no influence on cyt *c* level in *fro1* plants in our experiments (Figure 7C and Figure S2).

### 2.6. Reactive Oxygen Species Localization in fro1

**Figure 8:**
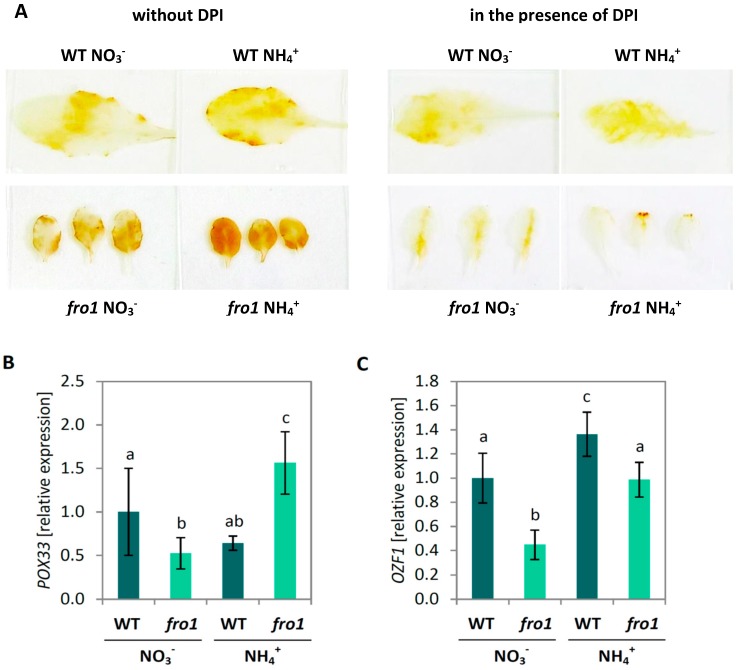
Extracellular ROS metabolism in *frostbite1* (*fro1*) or wild-type (WT) *Arabidopsis* ecotype C24 plants cultured on NH_4_^+^ and NO_3_^−^ as the only nitrogen source. (**A**) Visualization of leaf H_2_O_2_ content by 3,3-diaminobenzidine (DAB) staining in the presence or without diphenylene iodonium chloride (DPI). Representative results are shown. Transcript level for (**B**) peroxidase 33 (*POX33*) and (**C**) oxidation-related zinc finger 1 (*OZF1*). Values are the mean ± standard deviation (SD) of three biological and two technical replicates. Means with different letters are significantly different (*p* < 0.05) by ANOVA followed by Tukey’s test.

Hydrogen peroxide (H_2_O_2_) levels in plant tissues were visualized in situ via 3,3′-diaminobenzidine (DAB) staining. Brownish yellow color development in leaves of *fro1* was slightly more intense than in WT when grown on NO_3_^−^ as nitrogen source (Figure 8A). WT plants showed stronger coloration under NH_4_^+^ treatment, but *fro1* developed the most intense staining among all the analyzed leaves. The acute staining intensity implies that these plants had the highest H_2_O_2_ level in leaf tissues. Analysis of the presence of H_2_O_2_ in leaf tissues by DAB staining was performed simultaneously with a respiratory burst oxidase homolog (RBOH) and POX inhibitor diphenylene iodonium chloride (DPI) to eliminate apoplastic-generated ROS contents during the staining procedure. Color development in *fro1* leaves incubated with DPI was less intense than in WT plants under NO_3_^−^ nutrition (Figure 8A). On the other hand, under NH_4_^+^ nutrition, DAB staining in DPI-treated WT plants showed slightly reduced coloring, while the least coloration by DPI treatment was found in *fro1*. The difference in DAB staining with and without DPI enables the estimation of the amount of ROS accumulated in the apoplastic space. Results indicated that significant H_2_O_2_ accumulation in the apoplast was associated with NH_4_^+^ treatment, especially in *fro1* leaf cells (Figure 8A).

Next, we examined the expression of genes related to extracellular ROS metabolism. The expression of peroxidase 33 (*POX33,* AT3G49110), one of the POXs responsible for apoplastic ROS production, was slightly down-regulated in *fro1* plants, as compared to NO_3_^−^-treated WT plants. Ammonium nutrition induced *POX33* expression only in *fro1* plants (Figure 8B). Further, transcript level of oxidation-related zinc finger 1 (*OZF1*, AT2G19810), a plasma membrane protein involved in oxidative stress [64], was lower in *fro1* plants compared to WT under NO_3_^−^ treatment, but was stimulated in both genotypes under NH_4_^+^ supply (Figure 8C).

### 2.7. Changes in Mitochondria-Related Signaling in fro1

**Figure 9:**
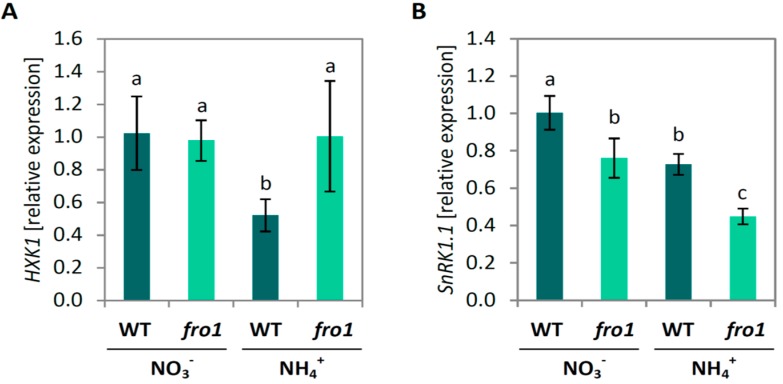
Marker genes for sugar signaling in *frostbite1* (*fro1*) or wild-type (WT) *Arabidopsis* ecotype C24 plants cultured on NH_4_^+^ and NO_3_^−^ as the only nitrogen source. (**A**) Transcript levels for hexokinase 1 (*HXK1*) and (**B**) sucrose non-fermenting 1–related kinase 1 (*SnRK1.1*). Values are the mean ± standard deviation (SD) of three biological and two technical replicates. Means with different letters are significantly different (*p* < 0.05) by ANOVA followed by Tukey’s test.

We determined transcript levels for marker genes of sugar signaling and retrograde signaling. First, we analyzed the expression of hexokinase 1 (*HXK1),* which is associated with the mitochondria, acts as a sugar sensor, and may regulate Glc-dependent gene expression [65]. Expression of *HXK1* in *fro1* mutants was similar to that in NO_3_^−^-supplied WT plants, regardless of the nitrogen source on which the mutants were grown (Figure 9A). Transcript level of *HXK1* decreased in WT plants when grown on NH_4_^+^. The expression of sucrose non-fermenting 1–related kinase 1 (*SnRK1.1*, AT3G01090), involved in sugar signaling pathways that responds to the availability of carbohydrates [66] was lower in *fro1* plants compared to WT under NO_3_^−^ conditions. Ammonium nutrition led to a decrease in *SnRK1.1* transcript level in both genotypes (Figure 9B).

## 3. Discussion

A major challenge for complex I mutant plants is to retain high energy levels required for maintenance and biosynthetic reactions. Accordingly, the complex I defect in *fro1* is associated with decreased biomass production in plants (Figure 1). In order to prevent the stunted growth phenotype, *fro1* plants strive to maintain constantly high ATP levels. In this regard, altered sugar catabolism might, to some extent, counteract the energy deficiency. The higher Suc and Glc (Figure 2A,B) contents in *fro1* plants may be used to produce energy in substrate-level phosphorylation, which confirms, for example, increased HXK activity (Figure 2C). In addition, NAD(P)H produced in the glycolytic pathway is channeled toward up-regulated type II dehydrogenases [47] to generate ATP in oxidative phosphorylation. Nevertheless, the lower ratio of ATP to ADP in *fro1* plants indicates that these plants cannot fully restore the energy-deficient status of cells [47]. 

### 3.1. Sugar Availability under Ammonium Nutrition May Limit Cell Wall Synthesis in WT but not in fro1 Plants 

**Figure 10:**
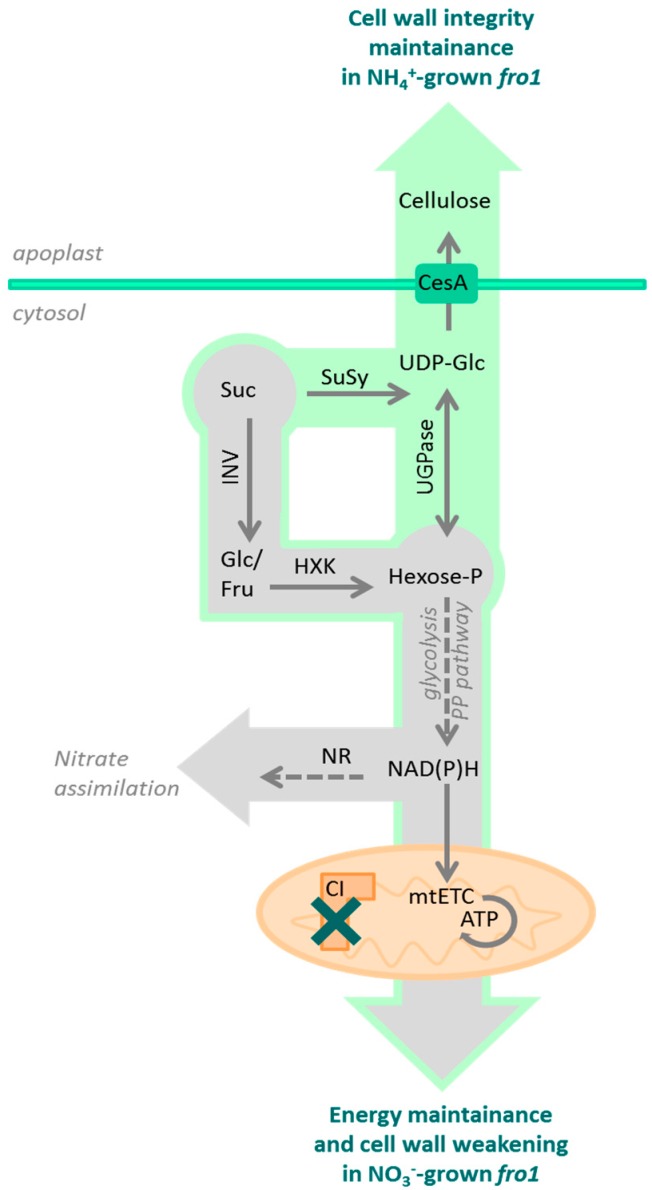
Carbohydrate metabolism in *frostbite1* (*fro1*) mutants lacking complex I (CI), when cultured on NH_4_^+^ or NO_3_^−^ as the sole nitrogen source. Sucrose, the major fixed carbon in plants, is channeled toward sugar catabolism via hexokinase (HXK) activity to generate hexose-phosphates (Hexose-P). Further, the glycolytic or pentose phosphate (PP) pathways provide reductants that can be oxidized in the mitochondrial electron transport chain (mtETC) to produce ATP. Alternatively to dissipation of reductants in the mtETC, high NAD(P)H expenditure is necessary for NO_3_^−^ assimilation catalyzed by nitrate reductase (NR). As indicated by grey arrows both energy fluxes are required to maintain the growth of *fro1* plants on NO_3_^−^. In contrast, when *fro1* is grown on NH_4_^+^, the reaction catalyzed by NR is omitted, resulting in a surplus of reductants. Therefore, the lower energy flux towards energy synthesis in *fro1* during NH_4_^+^ nutrition, allows sugars to be available for cell wall synthesis indicated as green arrow. Cytosolic sugars can provide a substrate for sucrose synthase (SuSy) or UDP-glucose phosphorylase (UGPase) to produce UDP-Glc, which is a precursor for cellulose synthesis. The cellulose synthetizing complex at the plasma membrane (containing cellulose synthase subunits, CesA) is responsible for the incorporation of carbohydrates into the cell wall.

Sugars are not only the ultimate source of energy and carbon skeletons for intracellular biomolecules but also provide the material used by plants to produce cell walls. While the plant cell is growing, an extensible primary cell wall is formed, the layers of which consist of cellulose microfibrils embedded in a matrix of cross-linked carbohydrates (hemicelluloses and pectin). Among the wall polysaccharides, cellulose, a polymer derived from β-1,4-linked Glc units, is the main load-bearing wall component [67,68]. The high input of sugars related to the energy-conserving phase in *fro1* plants might limit sugar availability for cell wall synthesis (Figure 10). In the present study, we detected lower cellulose synthase gene expression and decreased cellulose content in *fro1* plants, in particular, the expression of *CesA1, CesA3,* and *CesA6* (Figure 4B–D), which have been proposed to be connected with primary cell wall biosynthesis [60,69]. Similarly, as observed by Lee [46], the low incorporation of cellulose results in the generally thinner cell walls of these mutants (Figure 3C). Disturbed cell wall assembly might be a universal response in mitochondrial complex I mutants. In a proteomics study—an analysis of the functional context of altered proteins in the *ca1ca2* mutant line with impaired complex I—a major cell wall response was observed, although carbohydrate metabolism was affected to a lesser extent [22]. Alterations in sugar content have also been detected in another complex I mutant, *css1*, which was further characterized because of its lower cellulose synthesis [25]. Dysfunctional mitochondria of the *css1* mutant were proposed to compete with cell wall synthesis reactions for carbon, highlighting the branched pathways at the level of sucrose synthase (SuSy). UGPase and SuSy are involved in the synthesis of UDP-Glc in source tissues for cellulose production, and an unchanged UGPase protein level in *fro1* plants (Figure 2D) prevented restoration of the low cellulose synthesis in these mutants. It should be noted that UGPases have a dual function and might also promote the accumulation of free cytosolic UDP-Glc [70], and thus sugar breakdown instead of cell wall synthesis might be favored in *fro1* plants. In general, a high cell area to cell wall thickness ratio (Figure 3D) indicates that the cell walls in *fro1* plants might be weakened due to higher sugar flux towards catabolism. In *fro1* plants, NH_4_^+^ nutrition has the opposite effect on cell wall plasticity compared with that observed under control conditions, that is, WT plants have a lower cell area to cell wall thickness ratio in response to NH_4_^+^ supply (Figure 3D). This is because NH_4_^+^-grown plants are characterized by smaller cells (Figure 3A,B), and therefore the thin cell walls are relatively stronger compared to those of the small cells. In *Arabidopsis thaliana*, NH_4_^+^ nutrition has been found to increase cell wall firmness [58]. Despite a lower total cellulose content and *CesA* expression in WT plants during NH_4_^+^ nutrition (Figure 4), the cell wall thickness is not appreciably decreased as in *fro1* plants by the inactivation of complex I (Figure 3C). Substrate availability in the form of Suc and Glc, together with higher UGPase engagements (Figure 2A,B,D), might maintain cell wall synthesis at a level sufficient for small cells to grow. It can be assumed that NH_4_^+^-based changes in carbohydrate metabolism might compensate for the weak cell walls in *fro1* plants, thereby promoting better growth of these plants in the presence of NH_4_^+^. Therefore, in NH_4_^+^-grown *fro1* plants, a large proportion of soluble sugar (Glc and Suc, Figure 2A,B) might not be channeled to energy-producing processes (since HXK activity is decreased, Figure 2C), but rather toward cellulose synthesis due to higher UGPase engagement (Figure 2D). Consequently, in contrast to WT plants, the thickness of cell walls in *fro1* plants is not decreased in response to NH_4_^+^ nutrition (Figure 3C).

On completion of expansion, the structure of plant cells need to be strengthened, which is facilitated by the generation of a secondary cell wall that is mainly composed of cellulose, hemicelluloses, and lignin. The expression of *CesA4*, *CesA7*, and *CesA8*, which are genes associated with cellulose synthesis for secondary cell wall formation [71,72], were induced in *fro1* plants when grown on NH_4_^+^ and might be associated with a mechanism that compensates for the low cellulose deposition in these plants (Figure 4E–G). Plant growth is generally not directly related to cellulose availability but might be limited to some degree by cell wall rigidification. In the process of lignification, phenolic polymers are cross-linked to provide mechanical strength as a defense against different environmental stress conditions. In this regard, POXs have been found to catalyze the polymerization of a wide variety of small phenolic compounds [73]. For example, the cell wall-localized *POX64* and *POX72* isoforms have been shown to participate in this process [74,75], and we found that the expression of these two genes was increased in NH_4_^+^-grown *fro1* plants (Figure 5). Consistent with the previously observed higher expression of major CAD isoforms related to phenolic synthesis [76], we found that the expression of *CAD1* and *CAD5* was correlated with higher phenolic resources in cell walls (Figure 5C,E). Interestingly, despite higher substrate availability for lignification in NH_4_^+^-grown *fro1* plants, these plants had lower contents of lignin (Figure 5A) and showed lower POX activity [47]. Therefore, we did not expect the stiffening of the cell walls in *fro1* plants when treated with NH_4_^+^, which may therefore favor cell expansion. It should also be noted that POX activity might be involved not only in cell wall stiffening but also in contrasting processes such as cell wall loosening. In the hydroxylic cycle, POX can produce HO^−^ from the superoxide anion and hydrogen peroxide [77,78]. In response to NH_4_^+^ treatment, the selected *POX33* isoform showed an increased expression in *fro1* plants (Figure 8B), indicating that POX may play a role in non-enzymatic cell wall loosening, thereby enabling growth. Cvetkowska et al. [8] have proposed a relationship between defective mitochondrial functioning and processes occurring in the extracellular space associated with ROS-triggered signaling. Consistent with this supposition, we detected higher H_2_O_2_ levels in *fro1* plants, primarily within the apoplast (Figure 8A). The apoplastic ROS pool was even increased in *fro1* plants when grown on NH_4_^+^ (Figure 8A). A ROS-related response associated with the plasmalemma was also indicated by the induced expression of the marker gene *OZF1* in response to NH_4_^+^ in both *fro1* and WT plants (Figure 8C). Thus, we speculate that the ROS burst in the apoplastic space in response to NH_4_^+^ might activate signaling events [79,80].

The plant cell wall is an active structure that can respond to environmental cues, integrate signaling pathways, and regulate cell physiology and growth [81,82]. Perception of the integrity of cell wall cellulose can be ensured by dedicated cell wall sensor receptors such as kinases [83,84,85]. Cell wall remodeling in response to either NH_4_^+^ nutrition or a defect in complex I is reflected in the decreased expression of *FER* and *THE1* (Figure 3E,F). However, the effect of both sources of stress is to trigger a strong down-regulation of *FER* in *fro1* plants when grown on NH_4_^+^. Although *FER* is essential for expansion growth of cells, the biomass production of *fro1* plants is increased when cultivated on NH_4_^+^ (Figure 1). Mitochondrial dysfunction can in some cases induce tolerance against cellulose deficiency. In this regard, it has previously been shown that suppressed mitochondrial PPR-like protein induces retrograde signaling, resulting in a resistance to cellulose synthesis inhibition [86]. Accordingly, mutants can reconstruct weak cell walls and overcome growth suppression.

### 3.2. Fro1 Does not Show Significant Differences in the Pattern of Sugar Signaling

Sugars are probably the most important metabolites in the energy economy of living cells, and therefore cells need to have a precise system for monitoring sugar levels. Signalling pathways for sucrose, glucose, trehalose-6-phosphate, and fructose have previously been described [87,88,89]. HXK1 plays a dual role in cell metabolism, in addition to its enzymatic function of promoting hexose phosphorylation in glycolytic pathway, it can also act as a sugar sensor. Although *Arabidopsis* HXK1 is mostly associated with mitochondrial membranes, it is also expressed in the nucleus [87], where it forms a complex with specific subunits of other proteins and modulates the transcription of target genes. The expression level of *HXK1* has been demonstrated to be positively correlated with sensitivity to Glc [90,91,92]. In the present study, we found that expression of the *HXK1* gene in WT plants was decreased in response to NH_4_^+^ (Figure 9A). A lower expression of *HXK1* may be a mechanism whereby WT plants grown on NH_4_^+^ show a reduced sensitivity to increased levels of Glc. Additionally, it should be noted that HXK1-dependent Glc sensing is modulated by nitrogen availability [91] and in ammonium-stressed plants, nitrogen content is substantially increased (results not published). Although HXK activity in leaves of WT plants was not altered under NH_4_^+^ nutrition, it was relatively high in *fro1* plants under both growth conditions (Figure 2C). The energy metabolism of *fro1* plants depends largely on substrate-level phosphorylation, and this necessitates an up-regulated glycolytic flux in these plants. Indeed, HXK activity (Figure 2C) and soluble sugar content were increased (Figure 2A,B) in *fro1* plants. Furthermore, *HXK1* transcript levels remained unchanged and were at similar levels to those in NO_3_^−^-grown WT plants (Figure 9A), therefore the regulatory role of HXK1 in plants with dysfunction of the mtETC remains elusive.

The second well-described protein involved in sugar sensing in plant cells is the SnRK1 complex. This complex has kinase activity and is assumed to be regulated by sugar availability. However, recently, *SnRK1.1* has been recognized as playing a role in sugar-signaling, hub-regulating metabolism in response to changes in cellular energy status [93,94]. Among SnRK1-activated (and sugar-repressed) genes are those associated with catabolic pathways (cell wall, starch, Suc, amino acids, and protein degradation), which provide substrates for generating energy [95,96,97]. According to Baena et al. [95], SnRK1 senses stress-associated energy deprivation and reprograms metabolism to restore homeostasis and promote plant stress tolerance. *SnRK1.1* (also referred to as KIN10) is one of the catalytic subunits of the heterotrimeric SnRK1 complex in plants [98]. Surprisingly, we found that *SnRK1.1* transcript levels appear to decrease in response to NH_4_^+^ stress and mitochondrial complex I dysfunction (Figure 9B) when there is a cellular energy deficit [47]. Furthermore, SnRK1 activity was recently shown to be redox state-dependent [99]. Since the redox state of *Arabidopsis* leaf cells is increased in response to both mtETC dysfunction and NH_4_^+^ nutrition [47,57], we cannot exclude the possibility that this may induce SnRK1 activity despite lower transcript/protein levels.

### 3.3. Ammonium Nutrition Mitigates PCD Occurrence in fro1 Plants

Abiotic stress signaling or the energy status of cells can activate processes leading to PCD in plant cells. KOD [62] induces depolarization of the mitochondrial membrane, and constitutes an early step in plant PCD. Simultaneously, *KOD*-promoted PCD can be suppressed by the highly conserved survival factor *BI-1* which can delay the onset of PCD upon stress signaling [100,101]. Besides, a direct link between HXK1 activity and PCD has been proposed [91]. HXK1 inhibits PCD via binding to the voltage-dependent anion channel (VDAC) in plant mitochondrial membranes and inhibiting cyt *c* translocation from mitochondria in response to cellular stress [65]. In *fro1* plants, cyt *c* level was higher than in WT plants, (Figure 7C) however, a lower *KOD* transcript level and no changes in *HXK1* and *BI-1* expression (Figure 7A,B and Figure 9A) indicate that cell death in *fro1* plants under nitrate conditions (Figure 6) appears to be induced by other stimuli than the analysed genes. On the other hand, NH_4_^+^ nutrition has the opposite trend on marker gene expression, which correlates with unchanged cyt *c* abundance and the lack of lesion development (Figure 6 and Figure 7). Distinct differences in the molecular responses at the transcript and protein levels of *fro1* plants indicate that multiple pathways may be involved in mediating the progression or inhibition of PCD due to functional changes in mtETC or varying nitrogen supply.

Recently, Van Doorn [102] postulated the occurrence of two morphological classes of PCD: necrosis and vacuolar cell death. Necrosis is typically found under conditions of abiotic stress. Although necrosis is no longer considered to be an un-programmed process, it remains poorly characterized at the biochemical and genetic levels, and yet no associated molecular markers have been identified. Mitochondrial changes related to necrotic cell death include respiratory decline, the production of ROS, a decrease in ATP levels, and mitochondrial membrane permeabilization, most of which have been observed in *fro1* mitochondria [47]. Autophagy is an intracellular process involved in the vacuolar degradation of cytoplasmic components, and although it has yet to be determined whether autophagic pathways are required for the progression of vacuolar cell death, *ATG5*, one of the *ATG* genes that are essential for autophagosome formation, has recently been found to be involved in developmental vacuolar cell death of *Arabidopsis* [103]. In the present study, the induced expression of *ATG5* in *fro1* plants (Figure 7D) may thus indicate that lesions emerging on the leaf blades of *fro1* plants under NO_3_^−^ nutrition (Figure 6A) have the vacuolar cell death origin. Moreover, the decreased expression of *ATG5* which correlates with lack of lesions and fewer areas of dead cells in the leaf blades of NH_4_^+^-grown *fro1* plants, compared with those in plants grown under NO_3_^−^ nutrition (Figure 6), indicates that two different mechanisms underlie the responses of *fro1* plants to the nitrogen status. However, since the plants used in our experiments were long-term grown and PCD is a rapidly developing process, it is not possible to distinguish the exact morphological symptoms characteristic of both types of PCD.

## 4. Materials and Methods 

### 4.1. Plant Material and Growth Conditions

Experiments were performed on *Arabidopsis thaliana* plants of ecotype C24 (WT) and *frostbite1* mutants, which were derived through chemical mutagenesis as described by Lee et al. [46]. Plants were grown hydroponically using an Araponics system (Liège, Belgium) as described in Podgórska et al. [47]. The nutrient medium (according to [104]), containing 5 mM NO_3_^−^ or 5 mM NH_4_^+^ as nitrogen source was renewed twice a week. NO_3_^−^-treated WT plants were used as controls. Plants were grown for 8 weeks until they reached growth stage 5.10 according to [105]. The culture was conducted under an 8 h photoperiod at 150 μmol m^−2^ s^−1^ photosynthetically active radiation (PAR, daylight and warm white 1:1, LF-40W, Piła, Poland), day/night temperature of 21 °C/18 °C, and approximately 70% relative humidity.

### 4.2. Phenotype Analysis

Representative rosettes were photographed. Plant leaves were stained with 0.5 mg/mL Calcofluor White (Sigma Aldrich, Darmstadt, Germany) as previously described in Podgórska et al. [58]. The cross-section area of cells were determined using the Nikon A1R MP confocal laser scanning microscope (Nikon, Tokyo, Japan). Eight to 10 plants analyzed from each variant were randomly selected from 3 independent plant cultures. Cell size was calculated on micrographs using the Nis-Elements 3.22 imaging software (Nikon). The thickness of the cell walls was measured on micrographs obtained by transmission electron microscopy (TEM) (as previously described by [47]) according to Podgórska et al. [58]. The thickness of a double layer of cell walls was measured using the Image Processing and Analysis in Java software (ImageJ, v.1.51f, https://imagej.nih.gov/ij/). 

### 4.3. Lesions Identification

Selected leaves were photographed using a binocular to show lesion spots. The occurrence of spots on leaves was counted. The precise location of necrosis within leaf blades was analyzed using trypan blue staining [106]. The trypan blue solution was composed of 10% phenol, 10% glycerol, 10% lactic acid in 60% ethanol and 0.02% trypan blue [107]. Whole leaves were immersed in the trypan blue solution for 5 min at 35 °C; next, leaves were cleared with a distaining solution (40% methanol, 10% acetic acid, 10% glycerol) at 60 °C, and photographed. The staining intensity of trypan blue on leaves was quantified using ImageJ software.

### 4.4. Cell Wall Preparations, Cellulose, Lignin, and Phenol Content Assay

Cell walls were prepared from around 2 g of frozen leaf tissue as described by Solecka et al. [108]. The resulting precipitate containing the cell wall was air dried and used for cellulose and lignin determination. Cellulose content was measured via the colorimetric Anthrone protocol according to Updegraff [109]. Lignin content was determined by the acetyl bromide method [110] as described in Hatfield et al. [111]. The amount of phenolics bound to cell walls was measured using a method described in Forrest and Bendall [112], as described earlier in Solecka et al. [113]. Phenolics were released from the cell wall preparations by alkaline hydrolysis and their content was determined spectrophotometrically using the Folin reagent. 

### 4.5. Determination of Sugars and Protein Level

Soluble sugars were extracted as described in Szal et al. [114]. Glucose content was determined by the glucose oxidase-peroxidase reaction [115]. Sucrose concentration was determined after degradation to Glc and fructose. Protein level was measured as described by Bradford [116] using BSA as a standard.

### 4.6. Enzyme Activity Measurement and Protein Level Determination

Hexokinase activity was assayed according to the method described in Huber and Akazawa [117]. Protein extracts for enzyme activity determination and Western-blotting were prepared from 100 mg of leaf tissue which was homogenized with 2.5 volumes of extraction buffers.

Cytochrome *c* level determination in mitochondrial samples was done as described in Borysiuk et al. [63] and resulting bands were normalized on the basis of the mitochondrial marker protein voltage-dependent anion-selective channel protein 1 (VDAC1, Agrisera, Vännäs, Sweden, Figure S2). For other protein level analyses protein extracts (5 µL of protein extracts) (corresponding to 20 µg of protein) were separated in 10% sodium dodecyl sulfate-polyacrylamide gel electrophoresis (SDS-PAGE). Anti-UGPase [70], were used as primary antibodies (diluted 1:1000), and anti-rabbit antibodies (Bio-Rad, Hercules, CA, USA) were used as secondary antibodies. Immuno-blotting was performed according to standard protocols. Visualization was performed using a chemiluminescence kit (Clarity™ Western ECL, Bio-Rad, Hercules, CA, USA), and signals were detected using a Chemi-Doc imaging system (Bio-Ra). Bands (located at approximately 12 kDa for cyt *c* and 51 kDa for UGPase) were determined based on a pre-stained protein marker (Bio-Rad) as reference. Relative protein levels were quantified by densitometry analysis using Image-Lab 5.2. software (Bio-Rad).

### 4.7. Quantitative RT-PCR Analyses

Total RNA was extracted using a Syngen Plant RNA Mini kit (Syngen Biotech, Wrocław, Poland). DNAse digestion was performed using a RNase-free DNAse Set (Qiagen, Hilden, Germany). cDNA was synthesized using a RevertAid H minus first-strand cDNA synthesis kit (Thermo Fisher Scientific, Inc., Waltham, MA, USA) and RNAse H digestion was performed according to the procedure described in Escobar et al. [118]. The transcript levels were determined using iTaq Universal SYBR Green Supermix (Bio-Rad). Quantitative RT-PCR reactions were performed using a thermo cycler (CFX Content™, Bio-Rad) at 60 °C for annealing temperature. Reference protein phosphatase 2A (*PP2A*, AT1G13320, [119]) gene was used to normalize results. Transcript levels and qRT-PCR efficiency of genes were quantified as described in Pfaffl [120]. Results are expressed in relation to those in control plants. PCR primer pairs have been previously described for *FER* (AT3G51550), *THE1* (AT5G54380) [58], and *KOD* (AT4G22970) [62]. New primers were designed for: *OZF1* (AT2G19810), *CesA1* (AT4G32410), *CesA3* (AT5G05170), *CesA4* (AT5G44030), *CesA6* (AT5G64740), *CesA7* (AT5G17420), *CesA8* (AT4G18780), *SnRK1.1* (AT3G01090), *HXK1* (AT4G29130), *CAD1* (AT1G72680), *CAD4* (AT3G19450), *CAD5* (AT4G34230), *POX33* (AT3G49110), *POX34* (AT3G49120), *POX64* (AT5G42180), *POX72* (AT5G66390), *BI-1* (AT5G47120), and *ATG5* (AT5G17290) (Supplementary Materials Table S1), in which one sequence spanned always an exon–exon border if the gene had at least one intron.

### 4.8. Statistical Analysis

Results were expressed as means and standard deviations (SD) from 3 to 10 measurements taken from at least three independent plant cultures. One-way analysis of variance (ANOVA) and Tukey’s post-hoc test at *p*-values ≤ 0.05 were performed to analyze statistical significance of observed differences, using the Statistica 13.1 software (StatSoft, Inc., Tulsa, OK, USA).

## Supplementary Materials

Supplementary materials can be found at http://www.mdpi.com/1422-0067/19/8/2206/s1.

## Abbreviations

ATP	adenosine triphosphate
cyt *c*	cytochrome *c*
*fro1*	*frostbite1*
Glc	glucose
HXK	hexokinase
mtETC	mitochondrial electron transport chain
NADH	nicotinamide adenine dinucleotide, reduced
NADPH	nicotinamide adenine dinucleotide phosphate, reduced
PCD	programmed cell death
POX	peroxidase
ROS	reactive oxygen species
Suc	sucrose
TCA	tricarboxylic acid
UGPase	UDP-glucose pyrophosphorylase
WT	wild-type
