# Supplementary material for: Nitrogen Source Dependent Changes in Central Sugar Metabolism Maintain Cell Wall Assembly in Mitochondrial Complex I-Defective frostbite1 and Secondarily Affect Programmed Cell Death
Source: Int J Mol Sci. 2018 Jul 28;19(8):2206. doi: 10.3390/ijms19082206 (PMC6121878; doi:10.3390/ijms19082206)
Supplement: Supplementary file 1 [file ijms-19-02206-s001.pdf]

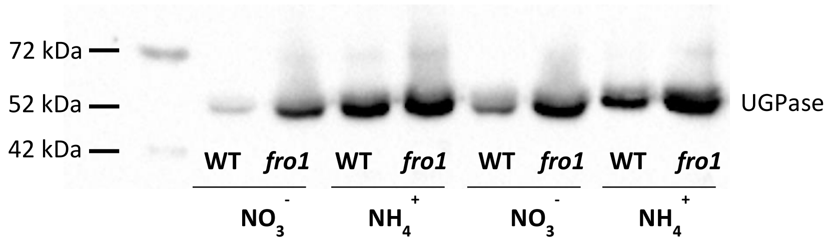

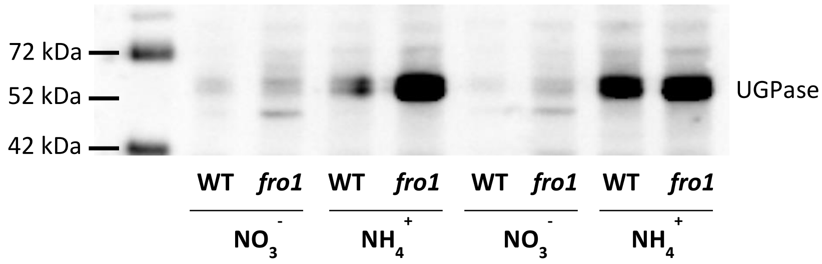

Figure S1. Protein level of UDP-glucose pyrophosphorylase (UGPase) in leaf tissue extracts of *frostbite1* (*fro1*) or wild-type (WT) *Arabidopsis* ecotype C24 plants cultured on NH_4^+ and NO_3^- as the only nitrogen source. Four biological replicates of the blot presented on Figure 2 are shown.

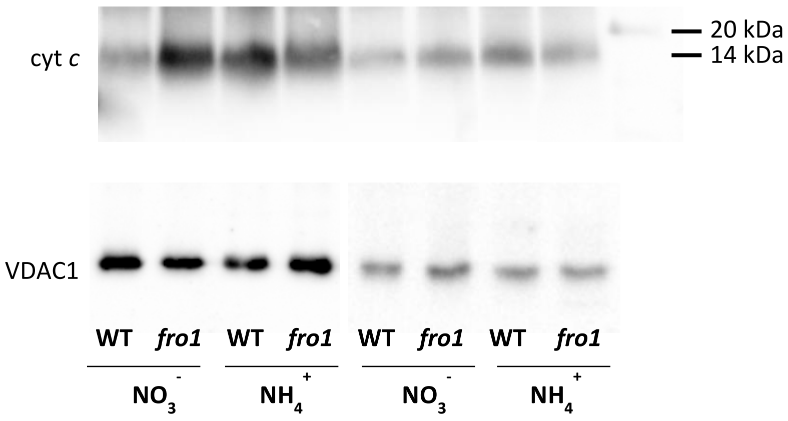

Figure S2. Cytochrome *c* (cyt *c*) protein level in mitochondria isolated from leaves of *frostbite1* (*fro1*) or wild-type (WT) *Arabidopsis* ecotype C24 plants cultured on NH_4^+ and NO_3^- as the only nitrogen source. Two biological replicates of the blot presented on Figure 7 are shown. Mitochondrial porin VDAC1 was used for protein level normalization.

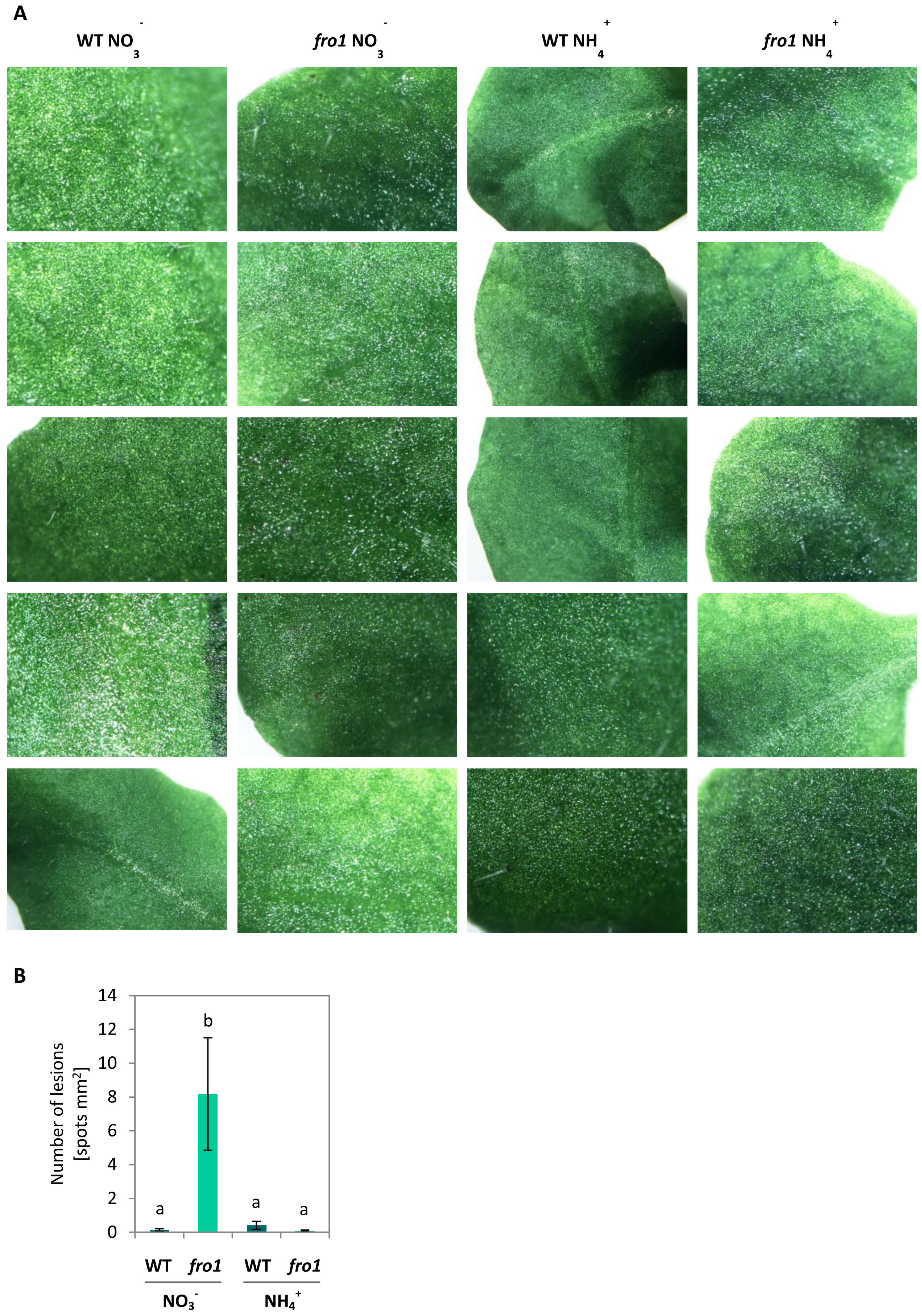

Figure S3. Lesions on leaves of *frostbite1* (*fro1*) or wild-type (WT) *Arabidopsis* ecotype C24 plants cultured on NH_4^+ and NO_3^- as the only nitrogen source. (A) Visualization of five biological replicates additional to the results presented on Figure 6. (B) Quantification of lesions on six representative leaves.

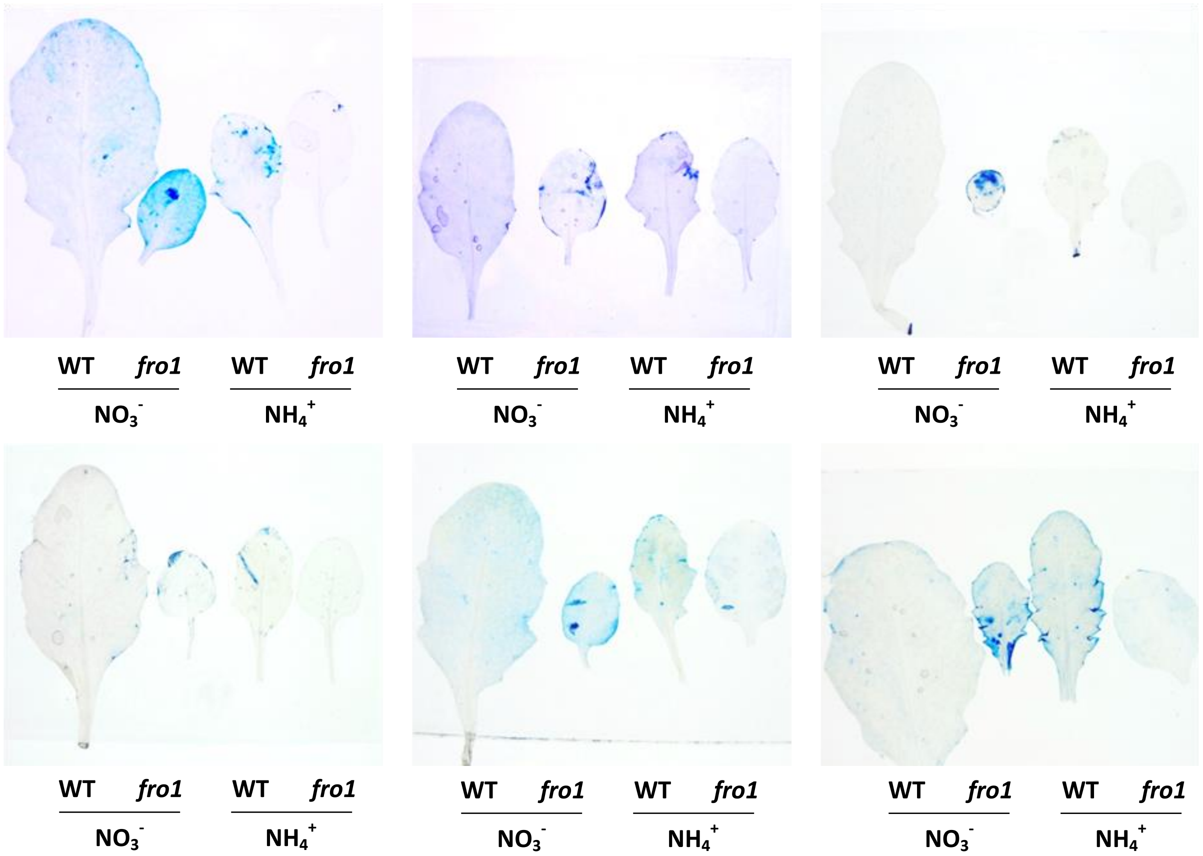

Figure S4. Necrosis development on leaves of *frostbite1* (*fro1*) or wild-type (WT) *Arabidopsis* ecotype C24 plants cultured on NH_4^+ and NO_3^- as the only nitrogen source. Six biological replicates of trypan blue staining additional to the results presented in Figure 6 are shown.

Table S1. List of qRT-PCR primers designed for this study.

Gene	AGI identification	Forward Primer	Reverse Primer
ATG5	AT5G17290	5’– TTCCTGTTCGGTTGTATGTTCG – 3’	5’– TCTTTCTTCACCACTCGTTTGC – 3’
BI-1	AT5G47120	5’– ATGTCCTCCTTATGAACACCAAA – 3’	5’– CAAGCCAGATGAAAGCAGTCC – 3’
CAD1	AT1G72680	5’– CTTGGTATGAGAATGCTCGCTG – 3’	5’– TGGGAATCACCTCTATGTTTGG – 3’
CAD4	AT3G19450	5’– CCTATGGTTCCTGGGCACG – 3’	5’– CGACTCCGACTACATCTCCTACG – 3’
CAD5	AT4G34230	5’– GCTTGGGAGGAAAGTGATAACG – 3’	5’– CGAGTCTCTCAAACGCAGTGT – 3’
CesA1	AT4G32410	5’– CATACGGTTTCTGGAGAGATTCG – 3’	5’– GACCCAAAGGACCTGATGTAGTTC – 3’
CesA3	AT5G05170	5’– CGAGCAGACAAGATACTTCAGGAG – 3’	5’– AGATACAGAGAGGCGTTCAGGTG – 3’
CesA4	AT5G44030	5’– GATGATTCAGGTGTATCTCGGCA – 3’	5’– GTAACCAGGACGCTTCTCTCTTG – 3’
CesA6	AT5G64740	5’– TAAAGGAAGTCCAAGAGTTGAAGGT – 3’	5’– TGCGACGAGAGATTGACATACC – 3’
CesA7	AT5G17420	5’– CAGCCCTTGGAGGAGCAGA – 3’	5’– CAAAGTTGAGGTTACAAAGATGGAT– 3’
CesA8	AT4G18780	5’– GGAAGATACGCCACCGAACA – 3’	5’– TAGGAATCACAACCGAAAGCAC – 3’
HXK1	AT4G29130	5’– CGAAGAAGCAGTTGGACAAGATG – 3’	5’– ATGGAAGATGTGATGACCTGAAGTT – 3’
OZF1	AT2G19810	5’– GCCGTCGTGTTTGTTTCTTTG – 3’	5’– CCCAGATTAGACCCGAGTGA – 3’
POX33	AT3G49110	5’– TGACTGCTTTGTTAATGGTTGTGA – 3’	5’– ACTGGAAATCCTCGGGCTG – 3’
POX34	AT3G49120	5’– GCTTTGTTAATGGTTGTGACGCA – 3’	5’– GGAAATCCCCGAGCCGAA – 3’
POX64	AT5G42180	5’– TGTTGCTCTCTCTGGAGGTCAT – 3’	5’– GAGGGGTTTAGTGTTGGGTCA – 3’
POX72	AT5G66390	5’– GACTGTTTCGTCAAGGGATGTG – 3’	5’– GGCTGAGTTTCTGTTAGGGTTT – 3’
SnRK1.1	AT3G01090	5’– CTTCAGTCTCGGGCTCATCC – 3’	5’– CGTCTCCAAAGTAGTTGTTATCGTG – 3’
